# Two-way adjustable double-knots intrascleral fixation and single sclerotomy looping technique: a novel minimal invasive adjustable intraocular lens fixation technique

**DOI:** 10.1186/s12886-023-03235-2

**Published:** 2023-11-23

**Authors:** Lulu Chen, Zaowen Wang, Lu Sun, Yongxiang Tang, Wenda Sui, Ailing Bian, Xia Zhang, Yong Zhong, Shunhua Zhang

**Affiliations:** 1 Department of Ophthalmology, Peking Union Medical College Hospital, Chinese Academy of Medical Sciences, No.1 Shuaifuyuan Wangfujing Dongcheng District, 100730 Beijing, China; 2 Department of Ophthalmology, The Affiliated Hospital of Zunyi Medical University, 563000 Zunyi, Guizhou China

**Keywords:** Adjustable, Intraocular lens, Surgical technique, Transscleral fixation

## Abstract

**Background:**

IOL fixation without capsular support presents challenges for surgeons. Although innovative techniques were developed to address subluxated IOLs, adjustable IOL fixation methods are seldom reported. We introduce a novel two-way adjustable double-knots intrascleral fixation combined with single sclerotomy looping technique for fixing intraocular lenses (IOL) or IOL-capsular bags.

**Methods:**

A bent 30-gauge needle threaded with 8 − 0 polypropylene was introduced into the eye. A gripping forceps assisted the haptic looping. Two overhand knots were made with 8 − 0 polypropylene thread. The knots were incarcerated into a scleral tunnel made by a 30-gauge needle, with two ends of the thread left at each side of the tunnel. The IOL was adjusted to the premium position with adequate tension by pulling either end of the threads. The study included 19 eyes with aphakia, subluxated IOL-capsular bags, or subluxated crystalline lenses. The mean followed up period was 18.9 ± 7.1 months with evaluations of uncorrected visual acuity (UCVA), intraocular pressure, slit-lamp examination, and swept-source optical coherence tomography of the anterior segment.

**Results:**

UCVA increased from 1.28 ± 0.74 at baseline to 0.44 ± 0.51 (logMAR) at final visit (*P* < 0.001). All IOLs were fixed well-centered. The mean IOL tilt was 3.5°±1.1°. Postoperative complications included transient IOP elevation (15.8%), hypotony (10.5%), and cystoid edema (5.3%) which resolved within 4 weeks.

**Conclusions:**

We presented a novel adjustable technique for IOL fixation, which stabilize IOLs by using an intrascleral double-knots structure. This technique minimized surgical manipulations by using a single sclerotomy looping technique without large conjunctival dissection and scleral flap creation. The technique offers a reliable and optimal IOL positioning and improved visual outcomes in patients undergoing scleral fixed IOL implantation.

## Background

Managing lens subluxation or intraocular lens (IOL)-bag subluxation is a challenging task for surgeons. Various surgical techniques have been developed, such as lens extraction coupled with vitrectomy and IOL fixation through methods like transscleral fixation, intrascleral fixation, or iris fixation [1–7]. The majority of these approaches necessitate extensive conjunctival dissection, scleral manipulations, and intricate needling and suturing techniques. Gore-Tex (W. L. Gore & Associates, Elkton, MD, USA) sutures and suturelss IOL fixation methods were developed later by some surgeons to reduce the incidence of suture related complications such as suture exposure, breakage, and erosion [8–12]. The basic idea is to tuck the haptics of a three-piece IOL into the scleral tunnels [13–16], but there is potential risk of IOL dislocation [17]. Yamane described a sutureless three-piece IOL intrascleral fixation method by making flanges on the haptics that were incarcerated in the scleral tunnel [18]. This method provided stable IOL fixation with small surgical trauma. However, the IOL positioning depends on the tucked haptic, making further adjustments difficult. In procedures involving suture fixation, surgical success is strongly reliant on properly centering the IOL and managing the tension of the threads effectively. Unfortunately, suture tension cannot be adjusted once the IOL is fixed using previously tied sutures or thread fastening [19, 20]. Any tilting or decentration of the IOL after fixation necessitates refixation, causing additional surgical trauma. In this paper, we introduce an innovative two-way adjustable double-knots (TADK) intrascleral fixation technique, combined with a single sclerotomy looping (SSL) method. This approach minimizes surgical trauma when looping the haptic and fixing the IOL or IOL-capsular bag by avoiding extensive conjunctival dissection and scleral manipulations. More importantly, the method optimized the IOL position by using an innovative two-way adjustable double-knots thread.

## Methods

### Subjects

This is a prospective case series including patients who underwent IOL or IOL-capsular bag intrascleral fixation from June 2019 to September 2022 at Peking Union Medical College, Beijing, China. The study adhered to the principles of Declaration of Helsinki and was approved by the Institutional Board of Peking Union Medical College Hospital. Informed consent was obtained from all participants. Consecutive patients with IOL-capsular bag subluxation, crystalline lens subluxation, and aphakia after pars plana vitrectomy for crystalline lens dislocation were included and the reasons for the lens subluxation were recorded. The exclusion criteria were retinal diseases requiring treatment, such as retinal detachment; preoperative intraocular pressure higher than 25mmHg while receiving treatment with eyedrops; scleritis. All the patients enrolled were evaluated with comprehensive preoperative and postoperative ophthalmic examinations including uncorrective visual acuity (UCVA), intraocular pressure, and slit-lamp examination of the anterior segment and fundus. History of previous systemic diseases such as Marfan’s syndrome, autoimmune connective diseases, or Weill-Marchesani syndrome were recorded.

All surgeries were performed by a single surgeon (ZWW) under peribulbar anesthesia. For cases with crystalline lens subluxation, phacoemulsification was performed before intrascleral IOL fixation. For cases with vitreous prolapse before or during surgery, anterior vitrectomy was performed to avoid vitreo-retinal traction.

The **single sclerotomy looping (SSL) technique** was applied to loop the haptics of IOLs or IOL-capsular bags. For IOLs with two haptics, two paracenteses were made 180 degrees apart. A 30-gauge needle was bent at the hub. An 8 − 0 polypropylene thread (Prolene, Polypropylene Suture; Ethicon, Johnson-Johnson, New Brunswick, NJ, USA) with curved needles at both ends was bisected. The free end (the leading end) of one half of the suture was threaded into the tip of the 30-gauge needle for approximately 3–4 mm. The 30-gauge needle was introduced into the eye 2.5 mm from the limbus at the fixation site. The tip of the 30-gauge needle passed through the closed loop of the haptic or pierce through the capsule covering the loop of haptic from back to front. A 23-gauge gripping forceps was passed into the anterior chamber through the paracentesis located opposite the fixation site. The 23-gauge gripping forceps grasped the thread out of the 30-gauge needle from the front of the haptic. The 30-gauge needle was retrieved a little bit and then forwarded to the front of the haptic. The 23-gauge gripping forceps delivered the leading end of the thread back to the tip of the 30-gauge needle and threaded for about 3 mm to create a loop around the haptic. After withdrawing the 30-gauge needle from the eye, the leading end of the polypropylene thread was externalized from the same sclerotomy at the fixation site. The same procedures were performed for the other haptic. For IOL with more haptics, similar procedures were performed with each haptic. For plate haptic IOL, the 30-gauge needle pierced the haptic at two diagonal sites to loop the haptic with similar procedures. For IOL with open loops covered by fibrosis capsular bag, the 30-gauge needle pierced the capsular bag at the midpoint against the haptic to loop the haptic.

**Fig. 1:**
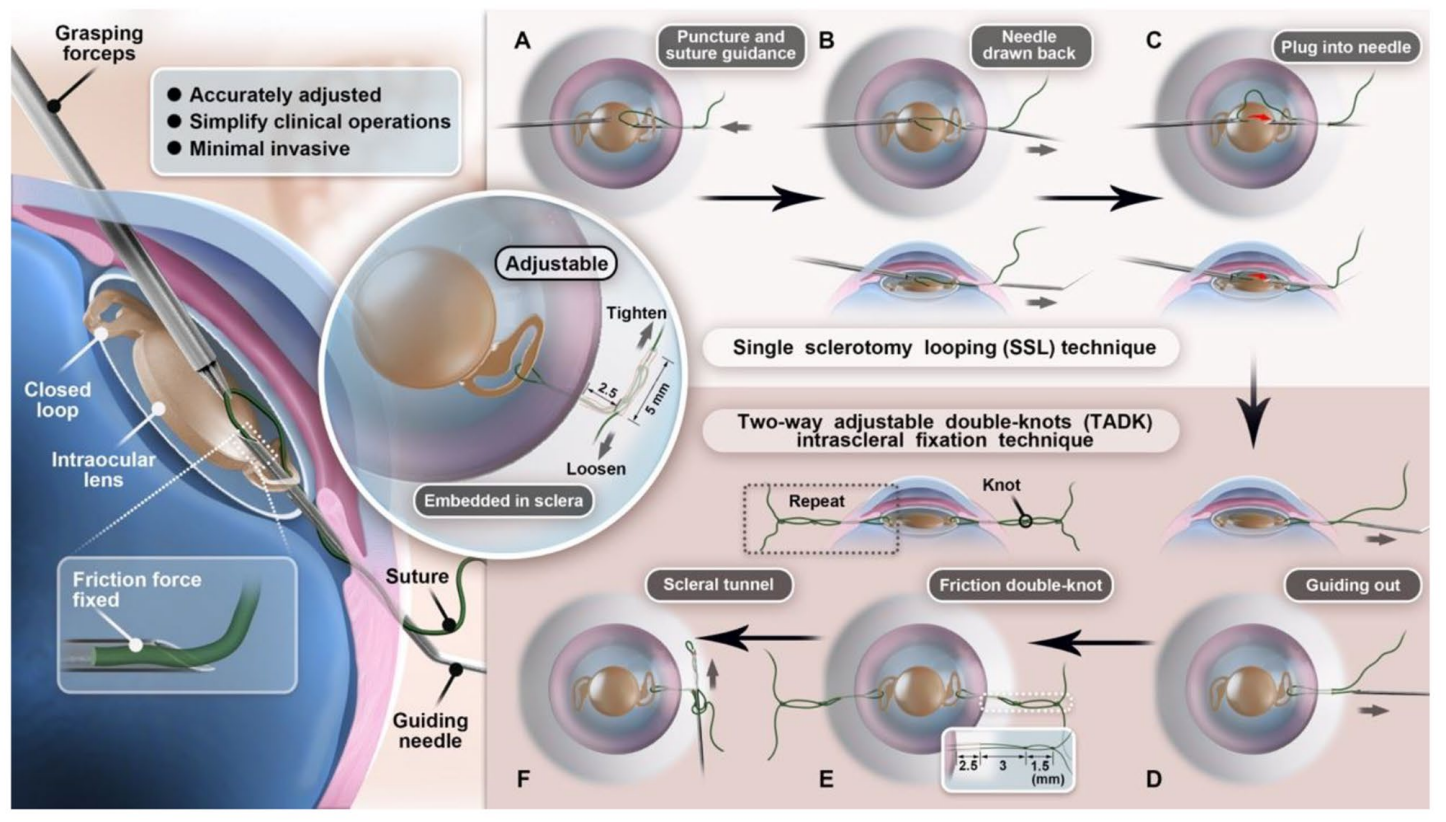
Schematic figure demonstrating surgical steps of two-way adjustable double-knots (TADK) intrascleral fixation with single sclerotomy looping (SSL) technique for IOL fixation. SSL(A-C). **A**, The 30-gauge needle with the leading end of 8 − 0 polypropylene thread was introduced into the eye 2.5 mm from the limbus at the fixation site. The tip of the 30-gauge needle passed through the closed loop of the haptic from back to front. **B**, The 23-gauge gripping forceps grasped the thread out of the 30-gauge needle from the front of the haptic. The 30-gauge needle was drawn back a little bit. **C**, The 30-gauge needle was forwarded to the front of the haptic. The 23-gauge gripping forceps delivered the leading end of the thread back to the tip of the 30-gauge needle. **D**, The 30-gauge needle was withdrawn from the eye and the leading end of the polypropylene thread was externalized from the same sclerotomy at the fixation site. TADK (e-f) **E**, Two 3-1-1 overhand knots with an interval of 1.5 mm were made with both ends of the thread 3.0 mm from the sclerotomy. **F**, The leading end of the thread was pulled to lead both knots entering the scleral tunnel and the other end of the thread was left at the beginning of the tunnel

The **two-way adjustable double-knots (TADK) intrascleral fixation technique** was performed for each externalized thread for fixation. A 3-1-1 overhand knot was made with both ends of the thread 2.0 to 3.0 mm from the sclerotomy. A second 3-1-1 overhand knot was made with both ends of the thread 1.5 mm from the first knot. The leading end of the thread was threaded into the 30-gauge needle for approximately 3 mm. The 30-gauge needle was used to create an intrascleral tunnel 1/3 to 1/2 of the scleral thickness in depth, from the sclerotomy 5 mm in length parallel to the limbus, and penetrated the conjunctiva. The leading end of the thread was grasped by a forceps and the 30-gauge needle was withdrawn. The leading end of the thread was pulled to lead both knots entering the scleral tunnel and the other free end of the thread was left at the beginning of the tunnel. A significant increase in friction can be felt when the knots entered the tunnel. The same double-knots procedure was performed for the externalized thread on the other haptic. The tensions of the fixation thread to centralize the IOL were adjusted by pulling either end of the threads. The thread was tightened when the leading end of the thread was pulled, the thread was loosened when the other free end of the thread was pulled. After the IOL centration was confirmed, the external ends of all threads were cut flush to the conjunctival surface (Fig. 1).

All patients were followed up 1 day, 1 week, 1 month, and 6 months or longer after surgery. Visual acuity, intraocular pressure, slit-lamp examination and dilated indirect slit-lamp biomicroscopy were evaluated at each visit. Postoperative complications were recorded. Swept-source optical coherence tomography (SS-OCT) (VG200; Svision Imaging, Ltd., Luoyang, China) of the anterior segment was measured 6 months or longer after surgery. The SS-OCT instrument used a central wavelength of 1050 nm (990-1,100 nm full width) and an A-scan rate of 200,000 per second. A standardized radial scan was performed after pupil dilation. The radial scan acquired 512 B-scan images with a length of 16 mm. The horizontal and vertical scans were used for IOL tilt measurement.

### IOL tilt measurements

**Fig. 2:**
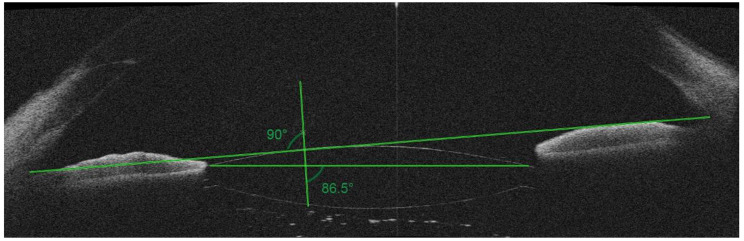
IOL tilt measured on SS-OCT image. The reference line was determined as the line passing through the iris-cornea angles on either side of the image. The angle between the line passing through the horizontal axis of IOL and the reference line was measured. The IOL tilt was measured in both the vertical and horizontal plans

We measured the angel of tilt of the IOL with methods reported by Yamane’s group [18, 21]. The reference line was determined as the straight line passing through the iris-cornea angles on either side of the image. The IOL tilt was measured in both the vertical and horizontal plans and the average of the IOL tilt of the two planes was defined as the mean IOL tilt (Fig. 2).

### Statistical analysis

Statistical analysis was performed using the software SPSS 25.0 (IBM, Chicago, USA). Continuous variables were summarized as mean and standard deviation. Normality of data was evaluated using Kolmogorov-Smirnov (K-S) test. Freidman test was used to compare visual acuity and IOP before and after surgery. Dunn test was used for post-hoc analysis between check points. A P value less than 0.05 was considered significant.

## Results

**Fig. 3:**
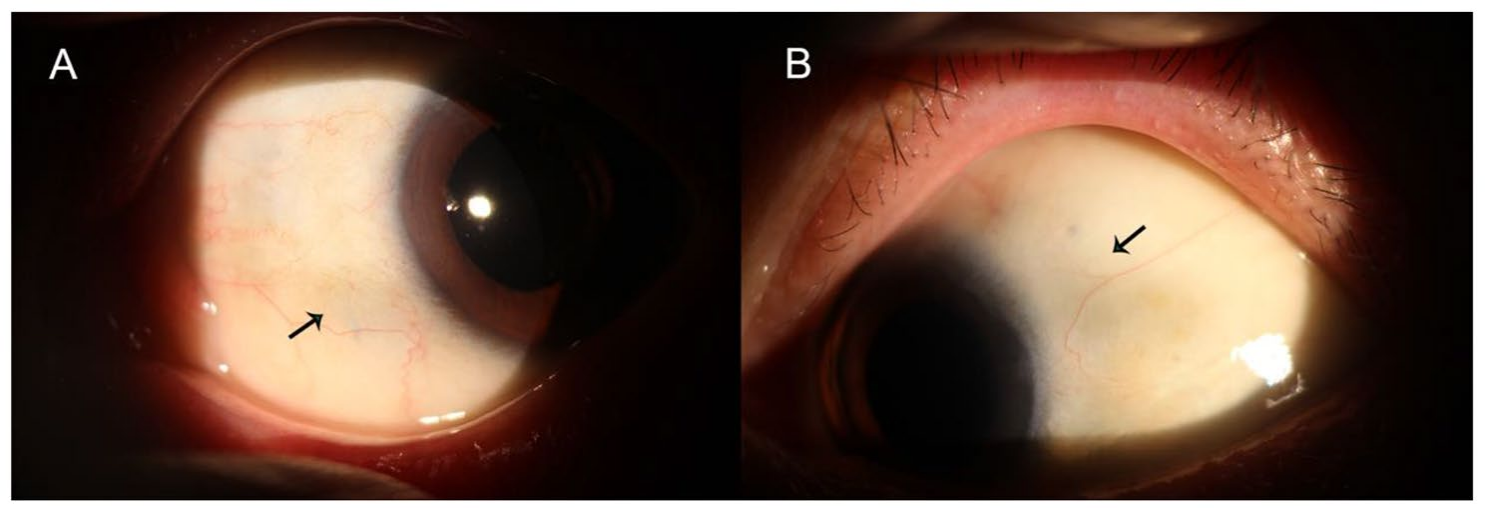
Postoperative slit-lamp photography of the left eye of a patient. The scleral tunnels were on the superior temporal and inferior nasal sclera. **A**, Slit-lamp image showing no conjunctival scarring and inflammation of the inferior nasal sclera. The arrow indicated the scleral tunnel. **B**, Slit-lamp image showing no conjunctival scarring and inflammation of the superior temporal sclera. The arrow indicated the scleral tunnel

**Fig. 4:**
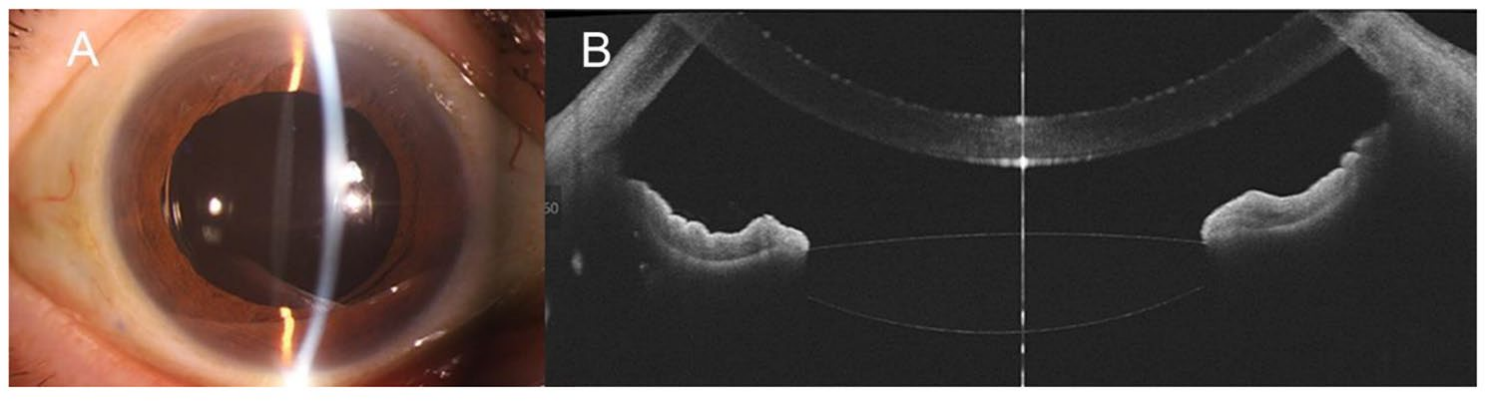
Postoperative evaluation of IOL position. **A**, Slit-lamp image showing a well centered IOL. **B**, Swept-source OCT of the anterior segment showing no tilt of the IOL

19 eyes from 17 patients (13 males and 4 females) were included in the study. The mean age was 61.9 ± 7.6 years old. All patients denied history of Marfan’s syndrome, autoimmune connective diseases, or Weill-Marchesani syndrome. Three patients had history of uveitis but denied history of autoimmune diseases. The baseline characteristics and IOLs used were presented in Table 1. The indications for surgery included IOL-capsular bag subluxation (n = 14), crystalline lens subluxation (n = 4), and aphakia after pars plana vitrectomy for crystalline lens dislocation (n = 1). Closed-loop haptic IOLs (RAO600C, Akreos MI60, CT ASPHINA 603P, SBL-3), plate haptic IOLs (CT ASPHINA 409), or in-the-bag open-loop haptic IOLs (Acrysoft IQ SN60WF) were used in the surgery. The mean follow up period was 18.9 ± 7.1 months (range 7–31 months). The preoperative logarithm of the minimum angle of resolution (log MAR) uncorrected visual acuity was 1.24 ± 0.74, which improved to 0.44 ± 0.51 at the final visit (P < 0.001). The mean preoperative intraocular pressure was 17.1 ± 4.5mmHg, and the mean postoperative IOP was 16.5 ± 4.1 mmHg (P = 0.999) at last visit. UCVA and IOP of each visit was summarized in Table 2. No conjunctiva scarring or inflammation was found with slit-lamp examination (Fig. 3). All patients had well-centered IOLs with mean IOL tilt of 3.5°±1.1° examined by SS-OCT (Fig. 4). Postoperative complications included a transient increase in intraocular pressure (> 25 mmHg) in 3 eyes (15.8%) which was managed by IOP lowering medications and turned to normal at 1 week postoperative follow up. Transient hypotony (< 10mmHg) was found in 2 eyes (10.5%) which returned to normal level after 3 days. Cystoid macular edema was observed in 1 eye (5.3%) 4 weeks after surgery and was treated with nonsteroid anti-inflammatory eye drops. The cystoid macular edema resolved after 4 weeks of treatment. No other postoperative complications, including hyphema, vitreous hemorrhage, endophthalmitis, choroid detachment, or retinal detachment were observed during the follow-up period.

**Table 1:** Demographics and baseline characteristics of patients who underwent IOL fixation with single sclerotomy looping and adjustable double-knots fixation technique

Total number of eyes	19
Total number of patients	17
Gender (male/ female)	13/4
Mean age (years)	61.9 ± 7.6
Follow up time (months)	18.9 ± 7.1
Causes of lens complications, n (%)	
IOL-capsular bag subluxation	14 (73.7%)
Retinitis pigmentosa	4
Trauma	4
Uveitis	3
Glaucoma	2
High myopia	1
Crystalline lens subluxation	4 (21.1%)
Pseudoexfoliation syndrome	2
Trauma	2
Aphakia after PPV for crystalline lens dislocation (Trauma)	1 (5.3%)
IOL type, n (%)	
RAO600C (Rayner, UK)	11 (57.9%)
Acrysoft IQ SN60WF (Alcon Inc. USA)	4 (21.1%)
Akreos MI60 (Bausch & Lomb, USA)	1 (5.3%)
CT ASPHINA 409 (Carl Zeiss Meditec, Germany)	1 (5.3%)
CT ASPHINA 603P (Carl Zeiss Meditec, Germany)	1 (5.3%)
SBL-3 (Lenstec Inc. USA)	1 (5.3%)

**Table 2:** Preoperative and postoperative UCVA and IOP.

	Preoperative	Postoperative-1 day	Postoperative-1 week	Postoperative-1 month	Postoperative->6 months	P
UCVA	1.24 ± 0.74	0.45 ± 0.48	0.43 ± 0.37	0.41 ± 0.35	0.44 ± 0.51	< 0.001
P1		0.011	0.015	0.002	< 0.001	
IOP	17.1 ± 4.5	16.3 ± 8.7	16.5 ± 7.6	16.8 ± 4.6	16.5 ± 41	0.834
P2		0.990	1.000	0.993	0.999	

UCVA, uncorrected visual acuity; IOP, intraocular pressure

P compared among measurements, Freidman test

P1 compared with preoperative UCVA, Dunn test

P2 compared with preoperative IOP, Dunn test

## Discussion

Managing crystalline lens subluxation or IOL-capsular subluxation is a complex task for surgeons, and numerous inventive techniques have been published to address subluxated IOLs. Concerns have been raised aiming at reducing surgical trauma and achieving stable premium IOL location. In this study, we reported a new technique of IOL looping and adjustable IOL intrascleral fixation.

The ab externo suture loop technique is a favored method that involves two needles shuttling back and forth through the eye to create a loop around the haptic [6]. Jin et al. modified this technique to create the loop intraocularly without retrieving manipulations [20]. However, several penetrations with a 27-gauge needle or guiding needle crossing back and forth in the eye are still required. The suture snare IOL fixation technique reported by Chee avoided intraocular needle shuttling by using a loop snare structure [12]. Sharing similar concept of belt and loop technique described by McCabe et al., our single sclerotomy looping technique needs no guiding needle and can be executed with just one penetration by a 30-gauge needle, minimizing the needle path inside the eye and reducing surgical trauma [22].

Sutureless and flapless intrascleral fixation techniques that avoid large conjunctival or scleral manipulations have been developed to minimize surgical trauma [18–20, 23, 24]. Methods using three-piece IOLs, such as the Yamane method, fix the IOL by creating flanges on the haptic. This technique, however, is not applicable to other types of IOLs. Alternative techniques fix the IOLs with intrascleral anchoring of thread, relying on a lumpy knot buried in the scleral tunnel to determine the IOL position. The friction between the knot and surrounding scleral tissue firmly holds the fixation thread and IOL. Jin’s method uses a 2-1-1 overhand knot [19]. We adopted this sutureless incarcerated knot concept to further minimize surgical trauma and enhanced it by creating two 3-1-1 overhand knots with a 1.5 mm interval, both incarcerated in a 5 mm scleral tunnel. The 8 − 0 thread’s knot size provides adequate friction and stability without obstructing the scleral tunnel. The tunnel length is sufficient for both knots to be incarcerated, forming an adjustable structure. By pulling either thread end, the thread tension can be adjusted, and the IOL position can be optimized. Slit-lamp imaging and SS-OCT were used to evaluate the IOL centration and IOL tilt and all IOLs were well centered in our study. IOL tilt after IOL fixation was reported to be 1.6° to 5.99° in different studies [18, 21, 25, 26], while IOL tilt of in- the bag IOL was reported to be 1.63° to 3.80° with different IOLs [27]. The mean IOL tilt was 3.5°±1.1° in our study, similar to those reported in intrascleral IOL fixation studies, showing the satisfactory IOL position of our method.

Techniques to provide optimized IOL position have been reported by some surgeons. Han’s group reported the in-and-out technique with addition of an internal fixation knot that facilitate the good centration and adequate tension of sutures [28]. Jin’s group reported a buckle-slide suture technique offering adjustable IOL fixation that reduces IOL decentration and tilt [29]. However, Jin’s method is only one-way adjustable, the tension of the tread can only be enhanced but not loosened. To our knowledge, we report the first two-way adjustable fixation technique. All IOLs were stable and well-centered without severe complications, and refixation was not needed during follow-up, demonstrating the technique’s safety and efficacy.

Besides, our technique effectively fixated an SBL-3 multifocal IOL, which demands higher accuracy in lens positioning. The lens remained well-centered throughout the follow-up period, suggesting the technique’s potential application in multifocal IOL fixation. Moreover, our approach is economically efficient, reducing patients’ financial burden, particularly those with multifocal IOL implantation.

This technique has several advantages. First, it doesn’t require special instruments; a 30-gauge needle, 23-gauge gripping forceps, and 8 − 0 polypropylene threads are easily accessible. The 8 − 0 polypropylene thread offers better tensile strength, stability, and lower cutting effect compared to 10 − 0 polypropylene thread [30]. Second, the technique minimizes patient discomfort by reducing surgical trauma and avoiding sutures, promoting faster wound recovery. Third, this method can be applied to various IOLs, including multifocal IOLs. Finally, the technique has fewer steps than other procedures and can be quickly mastered by surgeons.

No intraoperative complications were noted and the postoperative complications were similar to other methods of intrascleral IOL fixation. Postoperative transient increase and decrease of IOP was found in 15.8% and 10.3% of patients, and macular edema was found in 5.3% of patients. These complications resolved within a 4-week period. No severe complications including retinal detachment, vitreous hemorrhage, choroid detachment, IOL dislocation, or endophthalmitis were found. No exposure or erosion of the trimmed suture ends were detected.

The limitation of the study included a relatively small sample size. A larger cohort with more patients would better prove the efficacy of this technique. Besides, there is a lack of control group. Longer follow-up period was needed to evaluated the possible long-term complications of the technique and assess the long-term stability of the IOLs.

## Conclusions

In conclusion, we presented a novel two-way adjustable technique for IOL fixation, which stabilize IOLs by using an intrascleral double-knots structure. The single sclerotomy loop approach minimized surgical manipulations by using a single sclerotomy looping technique without large conjunctival dissection and scleral flap creation. The technique offers a reliable and optimal IOL positioning and improved visual outcomes in patients undergoing scleral fixed IOL implantation.

## Data Availability

The datasets presented in this study is available from the corresponding author upon reasonable request.

## Abbreviations

IOL: Intraocular lens
TADK: Two-way adjustable double-knots
SSL: Single sclerotomy looping
VA: Visual acuity
SS-OCT: Swept-source optical coherence tomography
UCVA: Uncorrected visual acuity
